# Gastrointestinal and Hepatobiliary Manifestations Associated with Untreated Celiac Disease in Adults and Children: A Narrative Overview

**DOI:** 10.3390/jcm13154579

**Published:** 2024-08-05

**Authors:** Herbert Wieser, Carolina Ciacci, Carlo Soldaini, Carolina Gizzi, Antonella Santonicola

**Affiliations:** 1 Hamburg School of Food Science, Institute of Food Chemistry, University of Hamburg, 20146 Hamburg, Germany; h.wieser2@gmx.de; 2 Gastrointestinal Unit, Department of Medicine, Surgery and Dentistry “Scuola Medica Salernitana”, University of Salerno, 84131 Salerno, Italy; carlo.soldaini@outlook.com (C.S.); carolinagizzi@libero.it (C.G.); asantonicola@unisa.it (A.S.)

**Keywords:** celiac disease, diagnosis, diseases, gluten-free diet, gastrointestinal and hepatobiliary manifestations, nutritional deficiencies

## Abstract

Celiac disease (CeD) is a chronic inflammatory disease of the small intestine, produced by ingesting dietary gluten products in susceptible people. Gluten causes an impairment of the mucosal surface and, consequently, an abnormal absorption of nutrients. Although malabsorption of essential nutrients is a major risk factor for various CeD-associated morbidities, genetic, immunological, and environmental factors also play an important role. The clinical presentation of CeD widely varies and can range from asymptomatic to full-blown symptoms due to the multi-system nature of CeD. The typical gastrointestinal (GI) manifestations of CeD include abdominal pain, diarrhea, bloating, and weight loss, but several hepatobiliary manifestations and a poor nutritional status have also been described. Currently, a gluten-free diet (GFD) is the only current evidence-based treatment that leads to the complete recovery of mucosal damage and the reversibility of its progression. Conversely, undiagnosed CeD might have severe consequences in children as well as in adult patients. This narrative overview aims to characterize the GI and hepatobiliary manifestations, nutritional deficiencies, and delayed pediatric development associated with unrecognized CeD in order to identify it promptly. Moreover, the role of GFD and how it could prevent long-term complications of CeD are described.

## 1. Introduction

Celiac disease (CeD) is a chronic immune-mediated disease characterized by small intestinal enteropathy. It is a unique autoimmune disease with an identified trigger: the gluten contained in wheat, barley, oats, and rye [1]. Originally considered a disorder exclusively affecting children, CeD has since been recognized as a disease affecting adults of all ages and has become a global disease estimated to affect about 1% of the global population [2]. Malabsorption of essential nutrients is a major risk factor for various CeD-associated morbidities; however, malabsorption alone cannot explain the pathogenesis, and immunological, genetic, and environmental factors play an important role. The clinical appearance of CeD is highly variable and can range from asymptomatic to full-blown symptoms due to the multi-system nature of CeD [3]. The typical gastrointestinal (GI) manifestations of CeD include diarrhea, abdominal pain, bloating, and weight loss. However, some patients report constipation, heartburn, or recurrent vomiting, which can lead to the wrong diagnosis of a functional GI disorder [4]. A wide spectrum of hepatobiliary diseases has also been reported, including an asymptomatic increase in liver enzyme levels, nonspecific hepatitis, nonalcoholic fatty liver disease, and autoimmune and cholestatic liver disease [5]. This wide clinical spectrum of CeD poses a challenge for its early diagnosis. Diagnosing CeD depends on the concordance of clinical, serological, and histopathological data. However, the diagnosis can be challenging and frequently overlooked. Most GI and hepatobiliary disorders associated with CeD can be treated with a strict gluten-free diet (GFD), but some of them are irreversible unless CeD is diagnosed in time.

This narrative overview aims to characterize the GI and hepatobiliary manifestations, nutritional deficiencies, and delayed pediatric development associated with unrecognized CeD in order to identify it promptly. Moreover, we also described the role of GFD and how it could prevent long-term complications of CED.

## 2. Methods

PubMed database searches were conducted for review articles published in English from 2013 to May 2024 using the keywords “c(o)eliac disease” in combination with “manifestations”. A total of 379 publications were retrieved, and 36 of them were selected. An additional 17 papers were identified by cross-referencing the retrieved reviews and personal files on CeD. Articles without abstracts, such as commentaries, letters, and conference papers, were excluded.

## 3. Esophageal Manifestations

Up until 30 years ago, several studies investigated the association of CeD with esophageal abnormalities such as gastroesophageal reflux disease (GERD) and eosinophilic esophagitis (EoE) [6] (Table 1). GERD is one of the most common chronic disorders found in the general population as well as in the CeD community, characterized by acid regurgitation, heartburn, increased salivation, and coughing. Several studies have demonstrated an increased frequency of reflux in patients with CeD. For instance, Nachman et al. evaluated the prevalence of GERD in two Argentinian cohorts consisting of 133 adult patients with CeD and 70 healthy controls [7]. At diagnosis, 30% of CeD patients had moderate to severe GERD, compared with 6% of controls. A rapid improvement of reflux symptoms was reported three months after initial treatment with a GFD. Vice versa, Mooney et al. showed that the prevalence of undiagnosed CeD in GERD patients (1.3%) was not enhanced compared with that of the general population [8]. Therefore, screening for CeD in GERD patients was not recommended.

EoE is an inflammatory condition of the esophagus presenting esophageal dysfunction and ≥15 eosinophils per high power field at esophageal biopsies. The affected esophagus does not contract properly, narrows, and develops rings or stenosis. An update until 2013 on the association between CeD and EoE, including 30 publications, revealed that most of the studies indicated that the prevalence of EoE in pediatric and adult subjects with CeD was around 10 times higher than that of the general population [9]. After 2013, further investigations on the relationship between CeD and EoE in children and adults showed much variability in the results [6]. Most studies indicated that the prevalence of EoE in CeD patients and the prevalence of CeD in patients with EoE were higher compared with the general population. Therefore, the existence of undetected CeD should be considered in individuals who have persistent EoE. Moreover, the role of food allergens in the pathogenesis of EoE is well established in scientific literature, as it is demonstrated how avoidance of certain food categories (milk/dairy, gluten/wheat, egg, soy/legumes, nuts, and fish/seafood) could lead to clinical and histological resolution [10].

In summary, most investigations revealed a higher rate of GERD and EoE in patients with active CeD. However, the pathophysiological mechanisms and the role of a GFD are still unclear, and further research is necessary.

## 4. Gastric Manifestations

Duodenal dysfunction and inflammation, present in active CeD, can have different adverse effects on the stomach [11]. For instance, abnormal gastric functions caused by untreated CeD may result in indigestion, burping, belching, sensations of fullness, nausea, and vomiting. When a GFD is introduced, these symptoms typically disappear, and patients reach a healthy state comparable to that of non-celiac individuals (Table 1).

Vomiting and nausea seem to be relatively specific symptoms related to gluten ingestion in CeD. To investigate the overall prevalence of these symptoms at CeD diagnosis and acute re-exposure during gluten challenge, medical data on 815 adult CeD patients were collected from the medical records and through supplementary interviews [12]. Twenty-eight patients (3%) presented with vomiting at diagnosis (data on nausea were not available). While on a GFD, 74 patients underwent a three-day gluten challenge (10 g/day). This short-term gluten challenge provoked vomiting in 10% of patients and nausea in 16% of patients.

Both children and adults with untreated CeD can present with gastritis and peptic ulcers. A five-year prospective study from Italy, including 213 adult patients with biopsy-proven CeD, revealed that at diagnosis, lymphocytic gastritis was present in 16% of patients, it was not associated with Helicobacter pylori infection, and it improved after GFD [13]. Both chronic active gastritis (32%) and chronic inactive gastritis (33%) were also frequently associated with untreated CeD but failed to respond to a GFD. To investigate the frequency of CeD in Turkish children with peptic ulcers, 74 patients underwent CeD-specific serological tests and duodenal histopathology [14]. CeD was diagnosed in 29% of the patients with peptic ulcers. Thus, there exists a high risk of CeD in children with peptic ulcers, and screening for CeD in patients with peptic ulcers, particularly those with no history of non-steroidal anti-inflammatory drug use, has been recommended. Investigations of the relationship between Helicobacter pylori infection and CeD have yielded conflicting results, which need further examination.

Altogether, chronic vomiting and nausea are frequent symptoms of untreated CeD and can be successfully treated with a GFD. Studies on the associations between CeD and gastritis or peptic ulcers and the effect of a GFD on these concomitant disorders are scarce, and, therefore, further research is necessary.

## 5. Intestinal Manifestations

The intestine is the main organ involved in the uptake of nutrients, electrolytes, and water, and simultaneously, it constitutes an essential barrier against the invasion of harmful microorganisms and toxins from the external environment. CeD is characterized by damage to the duodenal villous architecture and brush border, combined with the loss of mucosal enzymes and carriers. Moreover, the intestinal luminal surface is reduced from the area of a half badminton court to that of a sheet of paper [15]. The inflammation of the intestinal mucosa in untreated CeD can lead to a broad spectrum of intestinal complaints, such as chronic diarrhea, abdominal pain, bloating, and flatulence, which can be successfully treated with a strict GFD in most cases (Table 1). Today, most physicians are familiar with the association of CeD with these classical presentations.

One common CeD disorder in both adults and children is chronic diarrhea. Usually, patients refer to watery and loose stools occurring frequently or sporadically with intervals of regular stools. Food components (such as carbohydrates and fats) that the flattened mucosal surface cannot absorb cause the colon to produce water instead of absorbing it, which leads to diarrhea. Diarrhea can also be caused by bacterial overgrowth in the small intestine, which is indicative of active CeD. The so-called celiac crisis is a severe and potentially fatal clinical feature of CeD characterized by explosive watery diarrhea, metabolic and electrolyte disturbances, and shock, which requires hospitalization and parenteral nutrition. Previously, 15–20% of children with active CeD died of celiac crises. Today, it is rarely observed, mainly in children younger than two years of age but also in adults. Although uncommon, celiac crises should be considered in patients with unexplained chronic diarrhea [16].

Some CeD patients have periods of constipation, either in isolation or with diarrhea. Knowledge about the cause of constipation in untreated CeD patients remains insufficient. To determine the frequency of CeD in patients with chronic constipation, 1046 children were examined by CeD-specific serological tests and duodenal biopsy [17]. The results revealed a CeD ratio of 1:28, which corresponds to a CeD prevalence of 3.6%, which is much higher than that of the general pediatric population. In conclusion, the use of screening tests for CeD in children with conventional treatment-resistant constipation should be considered.

Undigested nutrients are fermented by bacteria in the small intestine and colon in individuals with active CeD, resulting in excessive gas production along with bloating, flatulence, and abdominal discomfort. One of the most reported symptoms of CeD in both adults and children is abdominal pain; in children, abdominal distension is a typical indicator of CeD. Lactose intolerance is caused by the loss of the enzyme lactase, which is typically produced by the brush border of the villi; it is additionally characterized by diarrhea, flatulence, and abdominal discomfort. Steatorrhea, which is caused by the malabsorption of fat, is characterized by oily, grayish feces that smell foul. Steatorrhea is considered more typical of active CeD than diarrhea. Therefore, the examination of stool fat after an in vivo challenge with gluten was one of the first diagnostic tools in the history of CeD. Altogether, classic intestinal symptoms such as chronic diarrhea, abdominal pain, bloating, and steatorrhea are well-known signs of unrecognized CeD in both children and adults, and today, physicians usually initiate the CeD-specific diagnostic approach.

There are sporadic reports of the occurrence of intussusception in CeD. The prevalence and natural history of intussusception in 150 newly diagnosed CeD children were prospectively studied by an Indian research group [18]. Intussusception, mostly involving the small intestine, was detected in 25% of children. Abdominal distension and hypoalbuminemia were found to be significantly associated with intussusception that resolved spontaneously on a GFD. All in all, serological testing for CeD has been recommended in children, especially with recurrent intussusception of the small intestine that occurs after the age of three in malnourished patients.

Several studies have suggested that CeD is associated with intestinal malignancies such as enteropathy-associated T-cell lymphoma, small bowel cancer, and colorectal cancer [19]. It has been assumed that a GFD may protect against or reduce the risk of malignancy in patients with CeD, but firm scientific confidence is still lacking. Studies that compared patients with CeD on a strict GFD with non-adhering CeD patients are scarce. In a cohort study from Italy, for instance, the risk of colon carcinoma in 1757 CeD patients was determined by Volta et al. [20]. During the follow-up period after CeD diagnosis (mean 18.1 years), six patients (0.34%) developed a colon carcinoma. The standard incidence ratio (SIR) was 0.29. Stratifying the risk for possible gluten intake, SIR dropped to 0.07 for CeD patients with strict adherence to a GFD. All in all, large multi-center cohort studies are needed to clarify the effect of a GFD on intestinal malignancies in CeD patients.

In summary, chronic diarrhea, abdominal pain, bloating, and flatulence are well-known complaints of unrecognized CeD in children and adults that can be resolved with a strict GFD in most cases. Intussusception and intestinal malignancies are rare, and their link to CeD is widely overlooked. They need more attention on the part of doctors and further research.

## 6. Pancreatic Manifestations

Several investigations have indicated an association between exocrine pancreatic insufficiency (EPI) and CeD, a less common cause of EPI itself [21] (Table 1). In a systematic review and meta-analysis, including six international studies, the prevalence of EPI in adult patients with active CeD versus those who had been on treatment with a GFD was examined by Jiang et al. [22]. All in all, 144 patients had newly diagnosed CeD, and 302 patients had CeD for at least nine months treated with a GFD. The individual rates of EPI in newly diagnosed patients ranged from 10.5 to 46.5% (mean 26.2%), and those of patients on a GFD ranged from 1.9% to 18.2% (mean 8%). Thus, patients with active CeD were significantly more likely to have an EPI compared with those treated with a GFD. Moreover, different studies are reporting a higher risk of both acute pancreatitis and chronic pancreatitis in CeD patients. CeD patients had worse outcomes and higher medical burdens compared with non-CeD patients [23].

## 7. Hepatic Manifestations

A significant minority (≈10%) of CeD patients have been diagnosed with several liver and biliary disorders, such as hepatitis B and C, autoimmune hepatitis, non-alcoholic fatty liver, liver cirrhosis or fibrosis, sclerosing cholangitis, and biliary cholangitis [24,25]. In rare cases, untreated CeD may lead to fulminant hepatitis and liver failure (Table 1). The pathogenesis underlying hepatitis injury in CeD appears to be multi-factorial and is still not completely defined [26]. A GFD improves the symptoms associated with CeD in many cases, and active screening for CeD is recommended in patients suffering from liver diseases with unclear etiology.

Most CeD patients with concomitant liver disorders have raised serum levels of the liver enzymes aspartate and alanine transaminase. Both adults and children are affected. For example, the level of alanine transaminase (ALT) in CeD and the effect of a GFD were examined in a cohort of US adult patients, including 463 subjects with biopsy-proven CeD tested at the time of CeD diagnosis and on a GFD (mean duration of 1.5 years) and 7789 matched non-CeD controls [27]. The results demonstrated that 40.6% of CeD patients at diagnosis, 24.2% of GFD-treated CeD patients, and 16.6% of controls had abnormal ALT. All in all, 79% of CeD patients with elevated ALT at diagnosis normalized ALT on a GFD. The prevalence, clinical course, and risk factors for hypertransaminasemia in 700 Italian pediatric CeD patients were studied by Benelli et al. [28]. ALT levels at CeD diagnosis were elevated in 27 children (3.9%). When retested on a GFD (>12 months), 24 patients (89%) normalized ALT levels.

Studies of 146 Indian patients with untreated CeD revealed that 26 of them (17.8%) had liver dysfunction [29]. Histological examination of liver biopsies revealed irregular sinusoidal dilatation (57.6%), steatohepatitis (15.3%), non-specific chronic hepatitis (11.5%), autoimmune hepatitis (7.6%), irregular perisinusoidal fibrosis, and changes of non-cirrhotic portal fibrosis (3.8% each). Follow-up liver biopsies after a GFD could be obtained in five patients; four of them showed resolution of the histological lesions.

Untreated CeD is considered a high-risk condition for fatty liver disease. For example, the examination of 221 newly diagnosed Italian patients with CeD demonstrated that 29.4% presented non-alcoholic fatty liver disease (NAFLD), while 14.5% met the criteria for metabolic-associated fatty liver disease [30]. Similarly, the association between CeD and primary biliary cirrhosis is well established. Both diseases share several features, including a higher prevalence in females, autoimmune comorbidities, and specific autoantibodies [31]. Reciprocal screening for both diseases is recommended since an early diagnosis with the appropriate treatment can improve the outcome of these patients. Not only the prevalence of NAFLD in CeD patients but also the prevalence of CeD in patients with NAFLD is increased compared with the general population. A large study of 3915 Egyptian patients with NAFLD showed that concomitant NAFLD and CeD were not uncommon; CeD was confirmed in 7.2% of patients with NAFLD [32]. Patients with concomitant NAFLD and CeD showed a clinical response to a GFD, but intestinal histological improvement was suboptimal.

## 8. Nutritional Status

Micronutrient deficiencies induced by malabsorption are common in individuals with untreated CeD. The degree of malabsorption depends on the size of the affected area of the small intestine and the length of time the disease has been active. While shortages of other water-soluble vitamins are less common, malabsorption of fat-soluble vitamins (A, D, E, and K), folic acid, and vitamin B12 is more frequent. The levels of minerals such as iron, calcium, magnesium, copper, zinc, and selenium can also be low in active CeD (Table 1). Patients suffering from CeD-mediated malabsorption are frequently afflicted by several disorders, such as delayed pediatric development, dental complaints, bone diseases, reproductive problems, and neurological manifestations [33].

Medza and Szlagatys-Sidorkiewicz recently summarized studies assessing the nutritional status of patients with CeD [34]. The prevalence of micronutrient deficiencies (vitamins and zinc) in patients with newly diagnosed CeD was as follows: D3, 0–32.7%; E, 0–88%; K, 0–21%; B6: 12.5–21%; B12, 19%; folate: 20%; and zinc, 3–67%. A retrospective study of 309 newly diagnosed CeD patients and 618 healthy controls revealed the following rates of vitamin and mineral deficiencies: D, 19% vs. 18%; B12, 5.3% vs. 1.8%; folate, 3.6% vs. 0.3%; zinc, 59% vs. 33%; and copper, 6.4% vs. 2.1% [35].

Most studies on the vitamin D status of CeD patients report a 25(OH) vitamin D deficiency at diagnosis that disappears when the patients go on a GFD, independently of any supplementation [36]. For instance, a case-control study of 131 Italian children with CeD showed a significantly lower mean 25(OH) vitamin D level at diagnosis compared with 131 healthy children (25.3 vs. 31.6 ng/mL) [37]. The percentage of children with vitamin D deficiency (<20 ng/mL) was significantly higher in CeD children than in healthy children (31% vs. 12%). Vice versa, individuals with vitamin D deficiency are at higher risk of developing CeD. A total of 200 Saudi adolescent girls with vitamin D deficiency (serum 25(OH) vitamin D < 50 nmol/L) were screened for CeD-specific serum IgA transglutaminase antibody levels [38]. Nine girls (4.5%) were seropositive, all with serum vitamin D < 12.5 nmol/L.

Iron-deficiency anemia (IDA) is one of the most common extra-intestinal manifestations in children and adults at CeD diagnosis. Anemia symptoms can appear as pallor, shortness of breath, chest pain, and a heart attack. Numerous studies confirmed the association between IDA and CeD by determining the frequency of CeD in patients with IDA or the frequency of IDA in CeD patients. In a systematic review and meta-analysis, including 18 studies and 2998 patients with IDA, the average prevalence of biopsy-confirmed CeD in patients with IDA was 3.2%, compared with around 1% in the general population [39]. Although IDA usually reverts to a GFD, some patients show persistent IDA, the mechanism of which is poorly understood.

All in all, vitamin and mineral deficiencies are common features of untreated CeD, and, therefore, the nutrition status of patients should be evaluated upon diagnosis of CeD. In cases of deficiencies, supplementation with missing micronutrients should be considered, and careful observation of nutritional parameters by professional healthcare providers of patients under a GFD may prevent ongoing nutritional deficits.

## 9. Pediatric Development

Generalized malabsorption is the most common cause of delayed pediatric development (Table 1). A cross-sectional Iranian study of 361 children with CeD revealed that 10.0%, 13.9%, and 22.4% had short stature, low body mass index, and low body weight, respectively [40]. Historically, weight loss has been considered a typical symptom of CeD. Recent studies, however, suggest that untreated CeD is also linked to overweight patients. A recent systematic review by De Giuseppe et al. evaluated the literature regarding the association between CeD and overweight/obesity in school-age children (aged 6–17 years) affected by CeD [41]. Overall, the results showed that the prevalence of overweight or obesity ranged between 3.5% and 20%, highlighting that the coexistence of CeD with overweight/obesity in children is not as uncommon as previously thought.

CeD is the most frequent cause of slow growth in children and is much more common than growth hormone deficiency. To study growth impairment in children with CeD, 113 infants who developed CeD within six years of age and 831 controls who did not develop CeD within six years were compared by measuring weight and length/weight ratio [42]. When the mean scores for weight and height were analyzed at the fourth month, one year, and six years of life, it emerged that children who developed CeD had a continuous significantly lower growth rate in weight score and length/height score than those who did not develop CeD.

Several studies have shown a relationship between untreated CeD and delayed dental maturation (DM). In a Saudi Arabian investigation, for instance, 104 children with biopsy-proven CeD and 104 healthy children were recruited to measure DM [43]. In the CeD group, 65 patients (62.5%) had delayed DM, and in the control group, only three participants (2.9%) had delayed DM. In conclusion, unexplained failure to thrive normally and delayed dental maturation indicate the existence of possible CeD and may be prevented by early diagnosis and the initiation of a GFD.

Delayed sexual maturation is also a known complication of untreated CeD. A Turkish two-center study aimed to assess the effect of CED on pubertal development [44]. Cohorts of 228 CeD patients (mean age 12.9 years) and 135 age- and sex-matched healthy controls (mean age 13.1 years) were studied using Tanner’s stages of sexual development (male genitals, female breast, male and female pubic hair). The results revealed that sexual maturation was significantly delayed in the CeD group for both girls and boys compared with the control group. Several studies dedicated to the effects of CeD on menarche onset resulted in contradictory results, and further investigations are needed to achieve statistically robust evidence.

In conclusion, slow growth rates and delayed dental and sexual maturation in children occur more often in CeD patients than in non-CeD controls, and early diagnosis of CeD and the introduction of a strict GFD in time may ensure normal growth and dental and sexual development.

## 10. Non-Responsive Celiac Disease

Non-responsive celiac disease (NRCD) is defined when symptoms, increased antibodies, or small intestinal damage persist after six to twelve months of a strict gluten-free diet. NRCD can be caused by dietary indiscretion, late healing, refractory CD (RCD), or another alternative diagnosis to CD.

Primary NRCD is characterized by a lack of response to a GFD, while secondary NRCD is characterized by an initial response to a GFD followed by the development of symptoms despite sustained GFD adherence.

There are several causes of NRCD [45]: immune ones such as common variable immunodeficiency, autoimmune enteropathy, Crohn’s disease, or collagenous sprue; infective causes such as Helicobacter Pylori, Giardia duodenalis, tropical sprue; small intestinal bacterial overgrowth; Whipple’s disease; or HIV. In some cases, the cause of NRCD may be related to medications such as angiotensin receptor blockers (sartans), methotrexate, mycophenolate, or immune checkpoint inhibitors (ICIs). In particular, there are a few case reports indicating that ICIs may also induce the onset of CeD [46,47,48] in predisposed individuals.

RCD is estimated to account for 8–23% of NRCD cases and has a documented incidence of 0.3–4% in CD patients. Individuals with RCD can be sub-classified as RCD Type 1 (RCD1) or Type 2 (RCD2), depending on the abnormal clonal expansion of a subset of small intestinal intraepithelial lymphocytes (IELs), which occurs in RCD2.

Patients with RCD2 are more likely to have constitutional symptoms, which include weight loss and nutritional deficits [49].

When additional symptoms like fever, bowel obstruction, night sweats, and gastrointestinal bleeding are present, RCD complications like ulcerative jejunitis (UJ), which is characterized by areas of multiple chronic ulcers that appear benign, and enteropathy-associated T-cell lymphoma (EATL), should be taken into consideration. In fact, it is reported that 33–67% of RCD2 cases progress to EATL [45,49].

## 11. Discussion

Nowadays, CeD is considered a heterogeneous disorder, both in its prevalence and in its clinical presentation. In fact, once labeled as an exclusively pediatric disease, it is now well established that it can affect every age group. Clinically, CeD is an extremely variable disorder, causing GI and extra-GI symptoms. Since the identification of gluten as a triggering factor by Willem K. Dicke in 1950 [50], a strict lifelong GFD has been introduced as the only effective treatment for CeD.

Within the digestive system, manifestations are various, ranging from those most canonically associated with CeD, such as chronic diarrhea, to others less well known to the global population. The inflammation and flattening of the duodenal mucosa in untreated CD, which lead to incomplete digestion and absorption of nutrients, are among the main mechanisms underlying the intestinal manifestations. On the other hand, the pathophysiological mechanisms of other manifestations, such as liver abnormalities, remain uncertain. Several hypotheses have been proposed and are currently debated in the scientific literature.

Most CeD-related conditions can be alleviated by a GFD, even if some of them may be irreversible if not treated early. For example, there is growth retardation in children. In the literature, there were even cases of severe liver diseases where transplantation could be avoided by early identification of CeD and treatment by a GFD [51]. The lack of adherence to the GFD, which is not uncommon [52], exacerbates pre-existing symptoms and may highlight new ones. On a strict GFD, children show faster and higher rates of symptom resolution as compared with adults [53], and GI symptoms show higher rates of improvement compared with extra-GI symptoms. Given the essential value of starting a GFD as early as possible, it is extremely important, not only for gastroenterologists and pediatricians but for every physician, to understand the broad spectrum of symptoms related to untreated CeD.

## Figures and Tables

**Table 1:** Gastrointestinal and hepatobiliary manifestations, nutritional status, and pediatric development associated with CeD and role of gluten-free diet on their clinical outcomes.

Organ	Manifestations	Gluten-Free Diet Effect
Esophagus	GERD	Unclear
	EoE	Potential benefit of food elimination diets (gluten/wheat) on clinical and histological remission.
Stomach	Vomiting, nausea Indigestion, burping, belching, sensations of fullness	Symptom resolution
	Gastritis and peptic ulcers	Unclear (gastritis and peptic ulcers)
Intestine	Chronic diarrhea Constipation Abdominal pain Bloating and flatulence Intussusception (unclear)	Symptom resolution
	Enteropathy-associated T-cell lymphoma, small bowel cancer	GFD may protect against or reduce the risk of malignancy (unclear)
Pancreas	Exocrine pancreatic insufficiency (EPI). Acute pancreatitis and chronic pancreatitis	Untreated ced patients have an increased risk of EPI compared with those treated with a GFD
Liver	Hypertransaminasemia autoimmune hepatitis Primary biliary cirrhosis NAFLD	Normalized alt and resolution of the histological lesions (in many cases)
Nutritional Status	Malabsorption: macronutrients, micronutrients, fat-soluble vitamins, folic acid, vitamin B12, and minerals	GFD may prevent ongoing nutritional deficits
Pediatric Development	Growth impairment, failure to thrive Low body mass index, low body weight Overweight patients Delayed dental maturation Delayed sexual maturation.	Timely introduction of a strict GFD may ensure normal growth, dental, and sexual development.

## Data Availability

The original contributions presented in the study are included in the article; further inquiries can be directed to the corresponding author.
